# Advancements in Mercury-Free Electrochemical Sensors for Iron Detection: A Decade of Progress in Electrode Materials and Modifications

**DOI:** 10.3390/s25051474

**Published:** 2025-02-27

**Authors:** Mahsa Motshakeri, Barbara Angoro, Anthony R. J. Phillips, Darren Svirskis, Paul A. Kilmartin, Manisha Sharma

**Affiliations:** 1 School of Pharmacy, Faculty of Medical and Health Sciences, The University of Auckland, Private Bag 92019, Auckland 1142, New Zealand; bnga207@aucklanduni.ac.nz (B.A.); d.svirskis@auckland.ac.nz (D.S.); 2 School of Biological Sciences, Faculty of Science, The University of Auckland, Private Bag 92019, Auckland 1142, New Zealand; a.phillips@auckland.ac.nz; 3 Surgical and Translational Research Center, Faculty of Medical and Health Sciences, The University of Auckland, Private Bag 92019, Auckland 1142, New Zealand; 4 School of Chemical Sciences, Faculty of Science, The University of Auckland, Private Bag 92019, Auckland 1142, New Zealand; p.kilmartin@auckland.ac.nz

**Keywords:** iron ions analysis, mercury-free electrodes, electrochemical sensors, modified electrodes, surface modification, voltammetry, potentiometry, amperometry, sample pretreatment

## Abstract

Efforts to quantify iron ion concentrations across fields such as environmental, chemical, health, and food sciences have intensified over the past decade, which drives advancements in analytical methods, particularly electrochemical sensors known for their simplicity, portability, and reliability. The development of electrochemical methods using non-mercury electrodes is increasing as alternatives to environmentally unsafe mercury-based electrodes. However, detecting iron species such as Fe(II) and Fe(III) remains challenging due to their distinct chemical properties, continuous oxidation-state interconversion, presence of interfering species, and complex behavior in diverse environments and matrixes. Selective trace detection demands careful optimization of electrochemical methods, including proper electrode materials selection, electrode surface modifications, operating conditions, and sample pretreatments. This review critically evaluates advancements over the past decade in mercury-free electrode materials and surface modification strategies for iron detection. Strategies include incorporating a variety of nanomaterials, composites, conducting polymers, membranes, and iron-selective ligands to improve sensitivity, selectivity, and performance. Despite advancements, achieving ultra-low detection limits in real-world samples with minimal interference remains challenging and emphasizes the need for enhanced sample pretreatment. This review identifies challenges, knowledge gaps, and future directions and paves the way for advanced iron electrochemical sensors for environmental monitoring, health diagnostics, and analytical precision.

## 1. Introduction

Iron is a multifunctional element with essential roles in biological, environmental, and industrial systems due to its redox-active nature. While necessary for life, iron can become a pollutant, secondary contaminant, and health hazard when present in high concentrations. In drinking water, iron levels above the World Health Organization’s guideline of 0.3 mg/L (5.36 μM) can lead to undesirable tastes, odors, and brown discoloration, indirectly impacting health and water quality [1,2]. Iron contamination in wastewater also disrupts ecosystems and significantly impacts marine biogeochemistry [3,4]. One consequence of iron in aquatic systems is its role in promoting the growth of photosynthetic algae, which increases the ocean’s capacity to absorb atmospheric CO_2_ and may contribute to climate regulation by potentially lowering global temperatures [5,6,7,8]. This influence on global climate underscores the need for accurate and precise monitoring of iron in water systems to safeguard environmental health, ecosystem stability, and climate balance. Iron analysis is also critical in agriculture and human health. Soil iron levels affect crop growth and quality, directly impacting food security and nutrition [9,10]. Iron is vital for oxygen transport, hemoglobin synthesis, and enzymatic functions in biological systems. Imbalances in iron levels, such as those contributing to anemia or iron overload, can cause tissue damage due to oxidative stress [11]. As such, rapid iron monitoring in biological fluids has important clinical implications [12,13,14].

To address this broad demand, practical and accessible methods for iron detection across various fields are needed. Traditional laboratory-based methods—including calorimetry, chemiluminescence, catalytic spectrophotometry, atomic absorption spectroscopy (with either flame (FAAS) or graphite furnace (GFAAS) atomizers), electrochemical techniques, flow injection analysis (FIA) (with chemiluminescence), voltammetry, and spectrophotometry, and inductively coupled plasma-mass spectrometry (ICP-MS) combined with optical emission spectrometry (ICP-OES) [13,15,16,17,18,19,20,21,22,23]—are widely used but can be expensive and complex. Electrochemical techniques such as stripping voltammetry, potentiometry, and amperometry provide cost-effective, user-friendly, and portable alternatives suitable for on-site detection. Traditionally, mercury-based electrodes were widely employed for their high sensitivity in iron ion detection. However, strict regulations have restricted its use due to mercury’s toxicity and associated environmental and health risks. These limitations have driven the development of mercury-free alternatives that offer comparable or superior analytical performance while being safer and more sustainable. However, conventional mercury-free electrodes often struggle to achieve the required sensitivity and selectivity for detecting iron species (Fe(II) and Fe(III)) in complex samples, primarily due to interference from co-existing ions, organic compounds, and matrix effects. This limitation is particularly problematic in trace and ultra-trace detection scenarios, where high sensitivity is essential.

In the last decade, significant progress has been made to design and develop modified mercury-free electrodes through modification strategies to enhance their performance and signal response [24]. These modifications, which often involve the use of nanomaterials, conducting polymers, ion-selective membranes, and novel ligands, have greatly improved the sensitivity and selectivity of iron sensors [25,26,27,28,29,30,31]. However, further validation is needed to ensure their reliability in complex sample matrixes. Despite these advancements, challenges remain to develop highly sensitive, selective, and interference-resistant mercury-free electrochemical sensors capable of trace and ultra-trace detection.

This review comprehensively summarizes the significant advancements in mercury-free electrochemical sensors for iron detection over the past decade. It significantly expands upon the last review in 2017, which provided a foundational overview but did not fully capture recent trends in mercury-free electrode modifications [24]. While previous work briefly touched on both mercury and mercury-free electrodes, our review aims to thoroughly examine a decade of advances in mercury-free electrode materials and surface modification strategies, which have enhanced iron sensor performance. This review compares electrochemical methods with conventional techniques. By identifying current research gaps and emerging challenges, this review seeks to guide future research directions for developing high-performance iron sensors with applications across environmental, health, and industrial sectors.

## 2. Conventional Techniques for Iron Analysis

Standard laboratory techniques for iron analysis include ICP-MS, ICP-OES, and atomic absorption spectroscopy (AAS) [13,22,23,32,33,34,35,36]. ICP methods offer high sensitivity for various elements with significantly low detection limits. However, these methods come with high operational costs, complex maintenance, and the need for technical expertise, making them best suited for centralized laboratories. Among these, ICP-MS stands out for its sensitivity and efficiency; it minimizes matrix interferences through isotope dilution without needing external standards or standard additions and can exclude isobaric interferences. ICP-OES, on the other hand, enables simultaneous multi-element detection (≥50 elements) over a wide concentration range (20 ppb–10,000 ppm), making it versatile for various sample types and applications.

Additional methods, such as FAAS and microwave plasma atomic emission spectroscopy (MP-AES), are commonly used for sequentially measuring a limited number of elements (six elements per day for FAAS and up to ten elements per day for MP-AES) at concentrations above 100 ppb. FAAS is suitable for analyzing a low to moderate number of samples (100–200 samples per day), whereas MP-AES can handle slightly larger sample volumes (300–500 samples per day). In comparison, ICP-OES, although more expensive, requires a lower sample volume for multi-element detection and offers faster analysis with lower detection limits, making it suitable for large sample sets (up to 2000–2500 samples per day). Due to their versatility, ICP and AAS methods are widely applied in environmental, geological, pharmaceutical, and biological fluid analysis and monitoring drinking water quality. For example, ICP-MS coupled with strong cation exchange liquid chromatography or capillary electrophoresis has been used to analyze iron species in clinical samples such as cerebrospinal fluids [13,14]. Despite these advantages, the weight, size, and high cost of ICP, AAS, and AES systems limit their use in land-based laboratories. Their restricted portability and susceptibility to matrix effects present additional challenges for on-site or real-time monitoring of iron in environmental and clinical samples.

Generally, conventional techniques vary in total iron detection and speciation (Table 1). ICP-MS [37] and ICP-OES [21] offer high sensitivity and speciation when coupled with separation methods. Similarly, if coupled with separation methods, AAS [38] and FAAS [39,40] can detect total iron and iron species. UV-Vis [41], colorimetric [42], and fluorescence [43] methods identify iron species via complexation or using fluorescent probes. Chromatographic techniques such as ion chromatography (IC) [44], capillary electrophoresis (CE) [45], high-performance liquid chromatography (HPLC) [21], and size exclusion chromatography (SEC) [46] separate iron species, protein-bound iron, and iron–ligand complexes. They can detect species when integrated with detection methods like ICP-MS or UV-Vis. Moreover, chromatographic methods are not suitable for total iron quantification.

In contrast, electrochemical methods offer a more practical alternative, as detailed in Table 2. They are inexpensive, lightweight, and portable, making them suitable for in-field applications where cost, accessibility, and rapid analysis are crucial. Although they may not provide isotopic composition data like mass spectrometry [47], recent electrode materials and surface modification advancements have enabled electrochemical sensors to achieve the selectivity and sensitivity needed for complex sample matrixes. This review will examine the advances made in non-mercury electrochemical sensors for iron species analysis, exploring how innovative surface modifications transform their performance across diverse applications.

**Table 1:** Conventional non-electrochemical techniques for iron analysis.

Techniques	Principle	Advantages	Disadvantages
ICP-MS [37]	Ionization of the sample in plasma, followed by mass spectrometry detection	Multi-element detection Ultra-sensitive, High throughput	Complex operation High cost Requires skilled personnel Spectral interferences
ICP-OES [21]	Excited atoms emit characteristic wavelengths, which are detected optically.	Fast with high sensitivity Multi-element analysis	Expensive instrumentation Matrix effects High argon gas consumption
MP-AES [48]	Microwave plasma excites atoms, producing emission spectra	Lower operating cost than ICP Multi-element detection Reduced argon consumption	Less sensitive than ICP-MS or ICP-OES Needs nitrogen gas supply Limited availability
AAS [38]	Absorption of light by ground-state atoms in a flame or graphite furnace	High specificity Good sensitivity Well-established method	Single-element analysis Slower compared to ICP methods Requires calibration
FAAS [39,40]	Flame-based atomization with light absorption measurement	Cost-effective Relatively simple Suitable for moderate iron levels	Lower sensitivity compared to GFAAS and ICP techniques Interference from matrix
UV-Vis [41]	Iron forms colored complexes with reagents and absorbance is measured	Simple Rapid Inexpensive Widely available	Limited sensitivity and selectivity Interference from other species and requires complexing reagents
Colorimetric [42]	Color change based on iron complexation with chromogenic reagents	Fast, easy to use, and portable Suitable for field testing	Prone to interferences Requires stable reagents Limited quantification accuracy
Fluorescence [43]	Iron ions quench or enhance the fluorescence of specific fluorophores	Rapid with high sensitivity Can be highly selective Suitable for iron binding studies in biological samples	Requires fluorophores Quenching effects can reduce accuracy Expensive instruments
Chromatography (e.g., HPLC [21], IC [44], SEC [46], and CE [45])	Separation of iron species based on interaction with the stationary phase, followed by another detection method	High resolution Suitable for speciation studies (e.g., IC and CE) when coupled with ICP-MS or UV-Vis Adaptable to complex samples	Complex sample preparation Expensive instruments Requires expertise Not suitable for total iron detection

**Table 2:** Comparison of electrochemical techniques for iron analysis.

Techniques	Principle	Advantages	Limitations
CV	Measures current response to a cyclic potential sweep	Characterizes electrode response Shows analyte redox behavior Fast scanning	Limited sensitivity for trace or ultra-trace detections
DPV	Applies small pulses with a linear potential ramp	High sensitivity Low background noise Trace metal detection	Requires optimized parameters
SWV	Combines pulses and staircase waveforms	High sensitivity Fast analysis Very low background noise Trace metal detection	Susceptible to matrix interference
AdSV	Preconcentration by adsorption, followed by stripping	Highly sensitive for trace iron detection No need for complexing	Requires precise preconcentration conditions
ASV	Preconcentration followed by oxidation scan	Excellent trace detection Suitable for Fe(II)	Requires careful electrode conditioning
CSV	Preconcentration followed by reduction scan	Suitable for Fe(III) High selectivity	Requires complexing agents
Amperometry	Measures current at a fixed potential over time	Simple setup Fast response For real-time monitoring	Limited selectivity Requires calibration
Potentiometry (Ion-Selective Electrodes)	Measures potential without applying current	No need for external redox reactions Simple	Less sensitivity compared to stripping methods
EIS	Measures impedance response over a range of frequencies	Provides insights into charge transfer and interfacial properties Can differentiate electrode modifications Suitable for studying sensor stability	Complex data interpretation Requires modelling for analysis

## 3. Electrochemical Techniques for Iron Analysis

### 3.1. Stripping Voltammetry Methods

Adsorptive stripping voltammetry (AdSV), anodic stripping voltammetry (ASV), and cathodic stripping voltammetry (CSV) are highly sensitive techniques for iron quantification and speciation [49,50,51]. These methods involve preconcentration, where ions accumulate on the electrode via adsorption or redox reactions, followed by stripping for detection. During the accumulation step, multiple interactions can occur at the electrode–electrolyte interface, including adsorption, complex formation, and precipitation of hydroxides, oxides, and salts, along with intermetallic reactions with electrode materials [52,53]. After accumulation and equilibration, ions are stripped from the electrode using voltammetric methods, such as linear sweep voltammetry (LSV), differential pulse voltammetry (DPV), or square wave voltammetry (SWV) [54,55,56], with peak height generated indicating analyte concentration. Factors such as the ion’s half-wave potential, scan rate, linear or pulse potential scan, stirring, electrode material, preconcentration time, and potential can influence the detection sensitivity. While competing reactions, saturation, sample pH, and hydroxide formation can limit the sensor performance.

AdSV enhances iron detection through non-electrochemical adsorption, particularly with carbon- [51,54] and bismuth-based [57] electrodes, as well as some modified electrodes [57,58,59,60], enabling high sensitivity at trace levels. ASV accumulates analytes via electrochemical reduction or adsorption, followed by positive potential scanning, making Fe(II) detection practical. However, optimal ligands or catalytic reagents are crucial for optimal results [49,55,56,61]. CSV, which involves electrochemical oxidation or adsorption followed by a negative scan, is well-suited for Fe(III) and total iron detection, with ligands, such as 1-(2-piridylazo)-2-naphthol (PAN) [50], 2-(5-bromo-2-pyridylazo)-5-diethylaminophenol (5-Br-PADAP) [58,62], and ω-thio nitrilotriacetic acid derivative [63], or without added ligands [64]. There is also a highly sensitive CSV method, competitive ligand exchange-CSV (CLE-CSV) using mercury electrodes, which involves complex ligand–metal interactions and requires precise calibration [65,66,67,68]. However, CLE-CSV falls outside the scope of this review due to the limitations of mercury electrodes. Instead, mercury-free CSV methods with iron-selective ligands are emerging, improving efficiency and simplifying analysis.

### 3.2. Amperometric Methods

Amperometry detects metal ions by applying a constant potential to a working electrode and measuring the resulting current over time, providing high sensitivity and real-time monitoring capabilities. This method is usually used for single-analyte detection in electrochemical sensors [69], particularly in biosensors, to assess enzyme inhibition by heavy metals [70]. However, amperometry is less commonly applied for iron analysis, with limited studies reported. For instance, Fe(III) has been quantified in wine using a carbon fiber microelectrode modified with ruthenium oxide hexacyanoferrate, achieving trace-level sensitivity [71]. Another approach used a rotating glassy carbon electrode modified with a conductive hydrogel composite, allowing micromolar Fe(III) measurements without requiring oxygen removal in acidic solutions [31]. This modified electrode effectively reduced Fe(III) to higher potentials, reducing sample preparation complexity.

Amperometry’s strengths include high sensitivity, simplicity, and suitability for in situ analysis, with enhanced selectivity when using surface-modified electrodes. However, it is susceptible to interference from dissolved oxygen and matrix components, which can destabilize signals. While surface modifications improve specificity, they can introduce variability and require precise calibration across sample types. Although amperometry shows potential for iron analysis, it is underutilized compared to its broader applications for other metals.

### 3.3. Potentiometric Methods

The potentiometric method measures metal ion concentrations by generating a voltage signal between a reference and a working electrode [72]. This technique offers advantages such as rapid response, low cost, minimal energy usage, and broad detection range for iron detection with high sensitivity and selectivity, particularly by modifying working electrodes with ion-selective membranes or iron-binding ligands or ionophores [25,73,74,75,76,77,78,79]. Advancements in potentiometric detection of iron used modified electrodes with nanomaterials or novel ligands/ionophores without using external membranes. Kaur et al. reported potentiometric and voltammetric Fe(III) detection with an impressive detection limit of 0.05 µM by modifying carbon paste electrodes with a Schiff-based ionophore [80]. These modifiers amplify detection capabilities by providing additional binding sites for iron ions, improving analytical performance sensors [80]. Similarly, Mohamed et al. quantified a wide concentration range (1–10 µM) of Fe(II) with a detection limit of 0.79 µM using a hybrid nanocomposite-modified carbon paste electrode [81]. However, potentiometric sensors can face challenges in achieving consistent performance and practical utility [72,82].

### 3.4. Electrochemical Impedance Spectroscopy (EIS)

EIS measures impedance at the electrode–electrolyte interface, making it a powerful technique for analyzing complex electrochemical reactions [83]. Recent advances exhibit EIS application in detecting metal ions, such as iron, due to its ability to monitor changes in the electrochemical properties of the interface in response to ion interactions. For instance, Shervedani and Akrami created calibration curves from different iron concentrations. They demonstrated that EIS enabled more sensitive and accurate Fe(III) analysis at lower detection limits compared to cyclic voltammetry (CV) and square wave voltammetry (SWV) using a self-assembled monolayer of deferrioxamine as a potent iron-binding ligand on a gold electrode [30]. Advantages of EIS include high sensitivity, the ability to detect subtle changes in impedance, and its non-destructive nature, which makes it suitable for real-time monitoring. EIS disadvantages include the need for sophisticated equipment, longer measurement times, and challenges in data interpretation, as the impedance spectra can be complex and require advanced fitting techniques for accurate analysis.

## 4. Mercury-Free Electrode Materials Used for Iron Electrochemical Sensors

Traditionally, mercury-based electrochemical sensors have been used to study metal ion speciation and multi-ionic analysis in complex samples at ultra-trace or trace levels [84], mainly through pioneering methods like polarography and voltammetry [85]. However, their use has declined due to significant drawbacks, including toxicity, environmental pollution, and regulatory restrictions. Mercury bioaccumulates in ecosystems, contaminates water sources, and forms toxic methylmercury, leading to strict regulations and a shift toward safer, mercury-free alternatives [84]. Additionally, mercury electrodes suffer from electrode fouling in complex matrixes, labor-intensive regeneration, and long-term instability, which further limit their applicability. In response, developing alternative, environmentally friendly electrode materials has gained momentum. Mercury-free electrode materials are designed to replicate or surpass the sensitivity and selectivity of mercury-based sensors while minimizing ecological and health risks.

Innovations in mercury-free electrodes and their modifications offer enhanced environmental safety, improved sensitivity, faster response times, and broader applicability. Electrode substrates for iron electrochemical sensors come in various sizes and designs, including bulk electrodes (macroelectrodes), microelectrodes, screen-printed electrodes (SPEs), paper, wafers, foam, and indium thin oxide (ITO). Bulk electrodes refer to unmodified conductive materials, such as glassy carbon, gold, platinum, graphite, and bismuth. These have well-defined electrochemical properties and exhibit planar diffusion, making them suitable for high-current applications. However, bulk electrodes often lack the necessary selectivity and sensitivity for trace iron detection in complex samples. To address their limitations, they can later be modified to enhance iron sensor performance. Microelectrodes with micrometer dimensions provide significant advantages over macroelectrodes, including enhanced three-dimensional (3D) mass transport, reduced capacitive currents, higher sensitivity, and steady-state diffusion responses. They are ideal for low-volume samples and miniaturized applications. SPEs are portable, cost-effective, and modifiable, making them suitable for disposable sensors and field-based applications. Wafers and ITO also provide stable, conductive platforms for advanced microfabrication. The choice of electrode substrate depends on analytical requirements, which highlight the importance of design in sensor performance.

Bulk electrodes and other electrode substrates can be modified with nanomaterials, conducting polymers, or ion-selective membranes to enhance selectivity by minimizing interference from other ions. Nanomaterials can provide a high selectivity and surface area, allowing for better differentiation between iron species and other co-existing ions. Additionally, modifications like molecularly imprinted polymers (MIPs) or the incorporation of specific ligands (e.g., chelating agents) can significantly improve the sensor’s selectivity by preferentially binding iron ions, reducing the effects of interfering substances.

Electrode modifications significantly improve electrical conductivity, mechanical strength, and chemical stability compared to unmodified electrodes [55,86,87]. The choice of synthesis materials and methods is also critical in defining the characteristics of the resulting structures [49,88,89,90], which increase active area, mass transport rate, and redox activity [90,91,92]. We have highlighted recent advances in these strategies throughout the review to emphasize their role in enhancing sensitivity and selectivity for iron detection in complex sample matrixes. As discussed in the following sections, these improvements make modified electrodes particularly well suited for iron-sensing.

### 4.1. Carbon Electrodes

Carbon electrodes are widely employed in various electrochemical applications, such as sensing iron. They are inert, easy to fabricate, cost-effective, with broad potential windows, excellent thermal and chemical stability, and biocompatibility [92]. Bulk carbon electrodes, such as glassy carbon electrodes (GCEs) [93], carbon paste electrodes (CPEs) [49,58], and graphite electrodes [94], along with carbon fiber microelectrodes [71], facilitate electron transfer and ensure efficient redox cycling of Fe(II)/Fe(III) species. Table 3 presents carbon-based electrochemical sensors that detect iron species with or without chemical modifiers. Carbon electrodes can be easily modified with various chemical modifiers, such as ruthenium oxide hexacyanoferrate (RuO_4_/Fe(CN)_6_) [71], and nafion [54], or specific iron-chelating modifiers, such as SMS-2 Schiff-based ionophore [12] and deferoxamine [95], to enhance selectivity and limit of detection (LOD) for iron species. Ruthenium oxide hexacyanoferrate-modified carbon fiber microelectrodes provided 0.22 µM LOD for Fe(III) in wine samples using amperometric methods [71]. Iron is the leading metal ion in wine, catalyzing oxidative chain reactions and affecting sensory attributes [96]; thus, its detection in the wine industry is crucial [71]. Iron concentrations greater than 6 mg/L in the presence of oxygen can lead to wine instability, odor, taste, color, and texture changes, which may impose further wine treatments [97,98]. Iron analysis in the beverage industry is also evident, as it can amplify the sweetness of sweeteners. So, knowing the iron level can lessen the consumption of sweeteners and lower costs in beverage production [99]. Applying nafion as a protective layer on electrode surfaces, such as graphite SPE, can improve LOD to as low as 15 nM [54]. It reduces electrode fouling and increases electrode resistance, mechanical stability, adhesion, and cation-exchange capacity [29,54,100]. Nafion also increases the capacitive double layer at the electrode–electrolyte interface to facilitate Fe(III) detection. Equations (1) and (2) illustrate the interaction of Fe(III) with the functional groups (R-SO_3_^−^) of nafion, where X represents the cation of a supporting electrolyte.

(1) $$3\left(R-SO_{3}^{-}X^{+}\right)+{Fe}^{3+}⇄\left(R-SO_{3}^{-}\right){Fe}^{3+}+3X^{+}$$

(2) $${\left(R-SO_{3}^{-}\right)}_{3}{Fe}^{3+}+e^{-}\to (R-SO_{3}^{-}{)}_{2}{Fe}^{2+}$$

Incorporating a chelating modifier like SMS-2, which contains a Schiff base linkage, into carbon-graphite ink on an SPCE (1:10 *w*/*w*) enhanced Fe(III) detection by DPV without the need for stripping methods [12]. This sensor successfully measured iron in human serum samples after pretreatment, including a 100-fold dilution, and pH adjustment to 7.0. Schiff bases are practical ligands for metal ion detection [101,102], featuring multiple binding sites due to their oxygen and nitrogen donor groups and an imine linkage, which can form complexes with Lewis acid cations. Upon coordination with an analyte, this ionophore generates an electrochemical signal. Another chelating modifier, deferoxamine, was immobilized on SPCE using benzophenone as an immobilizing agent and UV light treatment, which enhanced the electrodes’ stability for up to 20 weeks [95]. Deferoxamine has a high binding constant and can selectively form a hexagonal complex with Fe(III). This potentiometric sensor exhibited an LOD of 0.87 mg/L in standard iron solutions. When applied to various wine samples, the sensor strongly correlated with the results from a standard atomic absorption spectroscopy method [95]. However, chelating agents can be applied in sample solutions to improve the detection of Fe(II), Fe(III), or total iron by forming complexes. These ligands include triethanolamine(TEA) [93], 2-(5-Bromo-2-pyridylazo)-5-diethylaminophenol (5-Br-PADAP) [58], and *o*-phenanthroline [51]. Ustabasi et al. employed SPCEs without surface modifications, utilizing complexing and reducing agents in samples to detect Fe(III) and Cu(II) through adsorptive anodic stripping voltammetry [51]. Fe(III) was first reduced to Fe(II) using ferrocyanide as a reducing agent, and the resulting Fe(II)-*o*-phenanthroline complex was subsequently detected through oxidation during an anodic scan [51].

### 4.2. Gold Electrodes

Gold electrodes have been extensively exploited in the electrochemical detection of metal ions due to their remarkable conductivity, chemical stability, corrosion resistance, inertness, and ease of surface modification [30,63,103]. These properties make gold an ideal platform for creating selective and sensitive sensing interfaces, particularly in complex sample matrixes. Table 3 highlights the application of gold electrodes in detecting iron at nanomolar levels. A key advantage of gold electrodes is their compatibility with self-assembled monolayers (SAMs), formed through the chemisorption of sulfur-containing molecules such as thiols, sulfides, or disulfides. SAMs facilitate surface functionalization by immobilizing specific ligands or functional groups, enabling selective detection of metal ions [30].

Thiol-modified gold electrodes functionalized with iron-chelating ligands, such as deferrioxamine (DFO), have demonstrated high sensitivity and selectivity in detecting Fe(III) ions [30]. The binding of DFO to the gold electrode surface was achieved through the formation of a SAM, where 3-mercaptopropionic acid (MPA) anchors to the gold surface via Au–S bonds, and DFO was subsequently immobilized by covalent coupling to the carboxylic acid groups of MPA. This sensor achieved picomolar-level LOD for Fe(III) in aqueous solutions, with and without ferrocenemethanol (FcMeOH) as a redox mediator using CV, SWV, and EIS after 20 min of electrode holding at open circuit potential. It was applied to analyze various samples, including Ironorm capsules, Venofers ampoules, V.M. protein powder, ferrotitanium, and corn leaves, which exhibited consistency across the techniques. The electrode was regenerable via reverse CV scanning and stable for at least one week when stored under argon in 2 mM DFO containing ferrocenemethanol [30]. Similarly, other iron-binding ligands, such as ω-thio nitrilotriacetic acid derivative (ANTNA), were attached to gold electrodes through SAMs to enhance the detection of total iron at nanomolar ranges by DP-CSV [63]. Lower LODs for total iron concentrations were achieved by extending preconcentration times to 300 s. This sensor was reproducible, easily modified, and stored under argon gas. When applied to high-salinity marine water samples, no catalytic reagent or extensive sample pretreatment was required [63].

In another study, ferritin, an iron-binding protein, was covalently immobilized on gold electrodes functionalized with 6-mercaptohexanoic acid (C6) through SAMs to investigate and characterize iron release during electrochemical reduction in ferritin [104]. Phosphate was shown to be unnecessary for iron release, with Fe(H_2_O)_6_^2+^ released and subsequently oxidized to Fe(OH)_2_^+^, which adsorbed onto the electrode surface. These redox processes were not diffusion-controlled, with iron oxidation being kinetically faster than reduction. CV scans revealed a diminishing of anodic and cathodic peak currents, which suggested that the iron species diffused away from the surface after reduction. The redox reaction involved a two-proton, one-electron exchange that confirmed the identity of the reduced and oxidized iron species [104]. Finally, unmodified gold disk electrodes were employed to detect total and acidified dissolved iron. Using 5-Br-PADAP ligands in sample solutions and the DP-CSV method, these electrodes achieved nanomolar-level detection capabilities, further expanding the versatility of gold electrodes in iron detection applications [62]. This range of modifications and methodologies highlights the adaptability of gold electrodes as functional tools for designing sensitive and selective platforms to detect iron in various sample matrixes.

### 4.3. Platinum Electrodes

Platinum-based electrodes (PtEs) offer another reliable platform for the electrochemical detection of iron ions, distinguished by their low background current, a wide electrochemical potential window, and excellent catalytic properties. These features make platinum electrodes especially suitable for applications requiring high sensitivity and precision. However, the high cost of platinum remains a limiting factor that restricts its widespread use to specific applications. These electrodes are employed in various configurations, including wires, rotating ring-disk, rotating disk, Ag-alloy rotating disk, and platinum-Ag twin electrodes. Electrode surface modifications, such as coating with iodine, expanded the potential of platinum electrodes. Iodine-coated rotating disk platinum electrodes prevented oxidation/reduction reactions associated with oxygen and hydrogen adsorption and desorption and eliminated background interference in the working potential range (−0.25 V to +0.85 V) for Fe(II) detection [105]. This modification stabilized the electrode surface, reduced unwanted side reactions, and consequently enhanced the accuracy of measurements. Moreover, to increase the sensor response, reducing agents such as hydroxylamine were added to the sample [105]. In other approaches, using rhodamine-based dyes, such as rhodamine-dimethyliminocinnamyl (RC) [77] and rhodamine dimer (RD) [76] in sample solutions enhanced iron LODs through making iron complexes and leveraging their dual fluorescence and electrochemical properties (Table 3). All these synergistic strategies highlight the adaptability of platinum electrodes when combined with advanced chemical and material modifiers, offering robust platforms for iron detection across diverse sample matrixes.

**Table 3:** Iron electrochemical sensors based on carbon, gold, platinum, and bismuth electrodes.

Electrode	Iron Species	Reagent	Method	Calibration Range	LOD	References
Ruthenium oxide hexacyanoferrate/carbon fiber microelectrode	Fe(III)	None	Amperometry	10–210 μM	0.22 μM	[71]
GCE	Fe(II), Fe(III)	Triethanolamine	SWV	18–963 μM	38 μM	[93]
Deferoxamine/SPCE	Fe(III)	None	CV, potentiometry	1–10 mg/L	0.87 mg/L	[95]
SMS-2 ionophore/SPCE	Fe(III)	None	DPV	0.625–7.5 μM	0.93 μM	[12]
Nafion/Graphite SPE	Fe(III)	None	SW-AdSV	0.05–5 μM	15 nM	[54]
Bare SPCE	F(III)	*o*-phenanthroline and ferrocyanide	DP-AdSV	12.5–400 µg/L	3.74 μg/L	[51]
DFO/MPA/Gold disk electrode	Fe(III)	with (*) or without FcMeOH	CV	0.1–10 nM 0.3–100 nM *	0.1 nM 0.21 nM *	[30]
			SWV	0.1–10 nM 0.1–700 nM *	0.028 nM 0.034 nM *	
			EIS	0.1–700 nM 0.1–100 nM *	N/A 0.02 nM *	
ANTNA/SAM/Gold electrode	Total iron	None	DP-CSV			[63]
			Preconcentration time: 60 s	90 nM–1.4 μM	5.5 nM	
			Preconcentration time: 300 s	0.9–27 nM	0.2 nM	
Gold disk electrode	Total iron, dissolved iron	5-Br-PADAP	DP-CSV	0.01–1 μM	1.2 nM	[62]
Iodine-coated PtRD	Fe(II)	Hydroxylamine	LSV	0.4–100 ppm	0.07 ppm (~1.2 μM)	[105]
Graphite powder/Schiff-based ionophore/paraffine oil/PtE	Fe(III)	None	DPV	1–19 μM	0.05 μM	[80]
PtE	Fe(III)	RD	CV, DPV	15–350 μM	3.3 μM	[76]
PtE	Fe(II)	RC	CV, DPV	2–300 μM	0.16 μM	[77]
Sn-Bi alloy wires	Fe(III)	1-(2-piridylazo)-2-naphthol (PAN)	DP-CSV	1–900 nM	0.2 nM	[50]
Bi bulk annular band electrode	Fe(III)	Triethanolamine KBrO_3_	DPV	0.018–8.5 μM	5 nM	[106]

* Measurements were performed in the absence of FcMeOH as an external redox probe. N/A: Not applicable.

### 4.4. Bismuth Electrodes

Bismuth’s ability to form low-temperature alloys with heavy metal ions facilitates effective nucleation during the preconcentration step and offers detection sensitivities comparable to mercury electrodes [100]. Furthermore, bismuth exhibits advantageous electrochemical properties, such as excellent faradic behavior, partial insensitivity to dissolved oxygen, and good stability in alkaline media [107]. These attributes make bismuth electrodes a promising platform for iron sensing, particularly in stripping voltammetry techniques. Bulk tin–bismuth alloy wires were employed as working electrodes and achieved a low LOD of 0.2 nM for Fe(III) complexes with 1-(2-pyridylazo)-2-naphthol (PAN) in the detection range of 1–900 nM using DP-CSV method. This approach has proven effective in measuring total iron in environmental samples such as coastal rivers and seawater [50]. Similarly, bismuth annular band electrodes, when paired with catalytic reagents like potassium bromate (KBrO_3_) and triethanolamine (TEA), have shown a ten-fold enhancement in iron signal response without requiring a preconcentration step [106]. These bulk electrodes provide a cost-effective and reliable method for iron detection in diverse sample matrixes. Despite their advantages, bulk bismuth electrodes face limitations, particularly in their anodic potential range, which restricts their application in detecting metal ions at higher positive potentials. Optimizing the concentrations of catalytic reagents and carefully controlling background interference is essential to achieve consistent performance. To address these challenges, bismuth has also been incorporated onto electrode surfaces in nanomaterial forms to increase the number of active sites and facilitate interactions between the electrode/electrolyte interface and an analyte. Bismuth-based electrochemical sensors are generally non-toxic, stable in alkaline media, and provide excellent peak separation in stripping analysis, with partial insensitivity to dissolved oxygen. However, their low stability, and limited potential window in the anodic range restricts their use in detecting metal ions at higher positive potentials. Despite this limitation, their environmental compatibility and high sensitivity position them as promising alternatives for iron detection.

### 4.5. Nanomaterials and Composites-Modified Electrodes

Integrating nanomaterials and composite materials into electrode designs is a transformative approach in electrochemical sensor development [90,108,109]. These modifications leverage their components’ unique and synergistic properties to enable trace or ultra-trace quantification of analytes in electrochemical detection. Nanomaterials and composites enhance key electrode characteristics, including surface area, electrical conductivity, catalytic activity, mechanical strength, chemical stability, and analyte adsorption capacity [86]. The deposition of nanomaterials, such as carbon-based, metal-based, conducting polymer-based, and non-metallic-based nanomaterials, and their composites on bulk electrodes or other electrode substrates, improves sensing performance of iron electrochemical sensors, as highlighted in the following sections and summarized in Table 4.

#### 4.5.1. Carbon-Based Nanomaterials

Carbon-based nanomaterials, such as carbon nanotubes (CNTs), graphene, carbon black, graphene quantum dots, and carbon quantum dots, have been extensively used in developing electrochemical sensors for iron detection. Their high surface area, excellent electrical conductivity, and strong mechanical properties make them ideal candidates for enhancing sensor performance. Carbon nanotubes (CNTs) are classified into single-walled carbon nanotubes (SWCNTs, 0.4–2 nm), double-walled carbon nanotubes (DWCNTs), and multi-walled carbon nanotubes (MWCNTs, 2–100 nm). CNTs exhibit remarkable properties that make them highly desirable for electrode fabrication and are stable under harsh chemical and environmental conditions [118]. These materials were employed in developing electrochemical sensors to detect iron species [56,64]. CNTs can be either physically deposited on a GCE surface using an ethanolic solution of nafion, achieving a LOD of 0.71 μM [56], or grown directly on substrates where an iron catalyst was deposited in a chemical vapor deposition (CVD) furnace, resulting in a significantly lower LOD of 0.01 nM [64]. The substantial improvement in LOD with CVD-grown CNTs was attributed to their well-structured, vertically aligned morphology. This resulted in a stronger adherence to the substrate, enhancing electron transfer kinetics and providing an efficient sensing interface compared to physically deposited CNTs [64].

Graphene has gained significant attention within the carbon nanomaterial family due to its high surface area (2630 m^2^g^−1^), which is twice that of CNTs (1315 m^2^g^−1^). Its electrical conductivity exceeds that of copper and is 60 times higher than that of CNTs, making it a promising material for enhancing electron transfer in electrochemical sensors [119]. In a study by Sadeghi et al., the surface of a screen-printed carbon electrode (SPCE) was modified with graphene powders and piroxicam, a binding agent, which enabled the detection of iron with an LOD as low as 5.3 nM using a differential pulse catalytic adsorptive stripping voltammetry [117]. In another study, graphene synthesized by thermal exfoliation of graphene oxide (GO) in a nitrogen atmosphere, when combined with nafion, significantly enhanced Fe(III) detection by doubling the cathodic peak height after being drop-casted onto a platinum disk electrode surface [116]. The sensor showed a LOD of 0.08 ppb (1.4 nM) for Fe(III) after 180 s of pre-concentration.

Derivatives of graphene, such as GO and reduced graphene oxide (rGO), have attracted considerable attention in developing nanocomposite materials for electrochemical sensors due to their unique structural and electrochemical properties. Their functional groups, high surface area, and excellent conductivity enable effective integration with various nanomaterials, resulting in synergistic composites that enhance sensor sensitivity, selectivity, and stability. These materials have been employed in nanocomposites with metal or bimetallic nanoparticles, nanodendrites, biopolymers (e.g., polydopamine), and conducting polymers (e.g., polypyrrole) to develop advanced iron electrochemical sensors as summarized in Table 4 [89,112,113].

**Figure 1:**
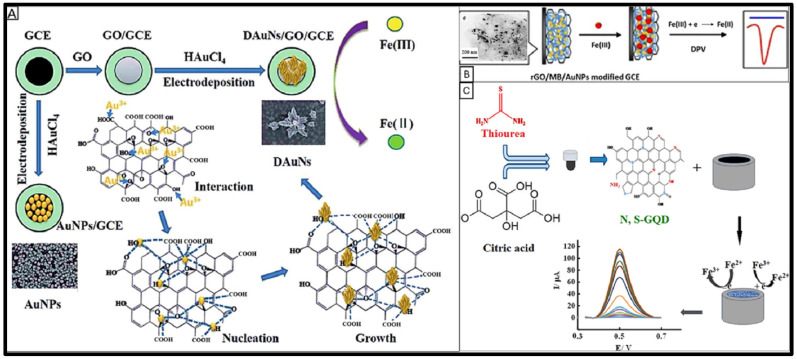
Electrode modifications with carbon-based nanomaterials: (**A**) Growth of dendritic gold nanostructures (DAuNs) on graphene oxide (GO)-modified GCE, which enabled Fe(III) detection at nanomolar levels. Reproduced with permission from [89]. (**B**) A GCE modified with a nanocomposite made of reduced graphene oxide (rGO), methylene blue (MB), and gold nanoparticles (GNPs) was used to detect Fe(III). Reproduced with permission from [111]. (**C**) Nitrogen and sulfur co-doped graphene quantum dots-modified glassy carbon electrodes (N, S-GQD/GCE) were used to detect total iron by SWV without a preconcentration step. Reproduced with permission from [27].

Oxygen-containing groups (carbonyl, carboxyl, epoxy, and hydroxyl) on functionalized GO improve Fe(III) detection by anchoring metal nanoparticles, facilitating iron reduction [89,90]. Figure 1A illustrates the optimum growth of gold nanodendrites on the large surface of a functionalized GO-modified GCE, which reduces the Fe(III) detection limits to as low as 1.5 nM. The presence of these oxygen-containing groups exerts a controlling effect on the electrodeposition and morphology of the gold nanodendrites [89]. Functionalizing GO with l-cysteine introduces mercapto groups on the surface that improve heavy metal absorption by forming chelates with metal ions, thus increasing nanoparticle loading and enhancing the electron transfer rate [120]. Zhou et al. drop-casted a nanocomposite of mercapto-functionalized GO and gold–bismuth nanoparticles on a GCE and detected Fe(III) by SWV. However, there were some discrepancies in the results that showed the inefficiency of the method [90].

Functionalizing chemically reduced graphene oxide (rGO) is essential to prevent irreversible aggregation and restacking caused by π-π stacking and van der Waals interactions, hindering rGO electrochemical performance [121]. Some iron electrochemical sensors have utilized rGO as the primary material in nanocomposites for modifying electrodes [55,111,112,113] due to its properties similar to pristine graphene and better conductivity than GO [122]. Using rGO as a support with a large specific surface area for the deposition of gold nanoparticles (AuNPs) improved the electrochemical reduction in Fe(III) in a complex form with a 5-Br-PADAP ligand in a buffer solution at −0.5 V potential, closed to the cathodic peak of free 5-Br-PADAP. Co-electrodeposition of rGO and AuNPs on a GCE in a single amperometric step created a nanocomposite with a low Fe(III) detection limit of 3.5 nM, enhancing sensitivity [113]. Incorporating gold nanoparticles and methylene blue (MB) into the rGO structure created a nanocomposite drop-casted on the GCE surface to accelerate electron transfer rates. In this nanocomposite, methylene blue acts as an electron mediator, anchors AuNPs, prevents rGO aggregation, and notably improves the cathodic peak of Fe(III) by about 10 times using DPV, as shown in Figure 1B [111]. Functionalization of rGO with reagents such as ionic liquids (ILs) with tuneable structures also improved the electrochemical performance of rGO [112]. ILs offer a wide electrochemical window, high ionic conductivity, unique thermal stability, excellent solubility, and notable biocompatibility [123,124]. Li et al. demonstrated that IL-rGO substantially increased the Fe(III) cathodic peak height by about 2.5 times and enhanced the detection sensitivity [112]. ILs act as reducing agents for GO and slow down the crystallization process of gold during electrochemical deposition owing to their high viscosity [112].

Self-polymerization of dopamine (a neurotransmitter hormone) in an alkaline condition can reduce GO and stabilize the resulting rGO, improving Fe(II) detection down to 0.9 μM LOD by DP-ASV [55]. Polydopamine acts as a biopolymer, which can introduce new functional groups, such as hydroxyl, amine, and imine, to the rGO, enhancing its electrochemical properties [55]. These groups strengthen electron transfer, improve electrode stability, and enable selective metal ion binding, making polydopamine-modified rGO a promising material for electrochemical sensors.

Another type of carbon-based nanomaterial is carbon black, which consists of amorphous carbon with moderate surface area, low mechanical strength, and limited conductivity. These materials are often used to develop cost-effective, scalable, disposable sensors. Carbon blacks enhance the electrocatalytic performance of sensors due to their high numbers of defect sites, nanoscale dimensions, and onion-like structures. Additionally, they can be quickly deposited onto electrode substrates through drop-casting methods to enhance iron detection [29,125]. Drop-casting carbon black dispersion developed an electrochemical sensing platform, followed by gold nanoparticles and nafion [29]. This platform demonstrated pronounced electrocatalytic activity towards Fe(III) detection in acid-pretreated serum samples by SWV, achieving a LOD of 0.05 mg/L (0.9 µM) in serum samples.

Graphene quantum dots (GQDs) and carbon quantum dots (CQDs) are zero-dimensional carbon nanomaterials that provide additional coordination sites and defects in their structures. They share similar properties with other carbon-based materials (e.g., graphene, carbon nanotubes, fullerenes) but are distinct due to their nanoscale size, high surface area, excellent conductivity, and photoluminescence properties [27,28]. GQDs can be applied to modify electrodes and immobilize ligands on the electrode surface. Co-doping GQDs with nitrogen and sulfur introduces electron-rich atoms into their structure. It facilitates charge transfer by increasing surface defects and active sites for electron transfer to Fe cations. Kalhori et al. reported the amperometric and SWV detection of Fe(III) using a GCE modified by drop-casting nitrogen and sulfur co-doped GQDs. The sensor achieved LODs of 0.23 nM and 1 nM for Fe(III) by amperometric and SWV methods, respectively, which demonstrated the sensor’s high sensitivity and selectivity (Figure 1C) [27]. However, Ma et al. designed a nanocomposite incorporating nitrogen-doped CQDs, β-cyclodextrin, and silver nanoparticles to simultaneously detect Fe(III) and Fe(II) using DPV. Despite the design, the method demonstrated limited sensitivity, as evidenced by the relatively high iron concentrations required for constructing calibration curves [28].

#### 4.5.2. Metal-Based Nanomaterials

Metal-based nanomaterials are powerful modifiers for electrochemical sensors due to their high conductivity, catalytic activity, chemical stability, and tuneable surface properties [126,127]. Their versatility stems from various morphologies (e.g., nanoparticles, nanorods, and nanodendrites) and compatibility with composite materials, which enhances electron transfer and detection sensitivity. Bimetallic and metal oxide nanomaterials offer synergistic benefits, including enhanced catalytic efficiency and stability [90,126]. These nanomaterials are integrated into nanocomposite structures through strategies such as electrochemical deposition, self-assembly, drop-casting, co-fabrication with polymers or carbon-based materials [111,113], as well as the formation of bimetallic alloys and core-shell architectures.

**Figure 2:**
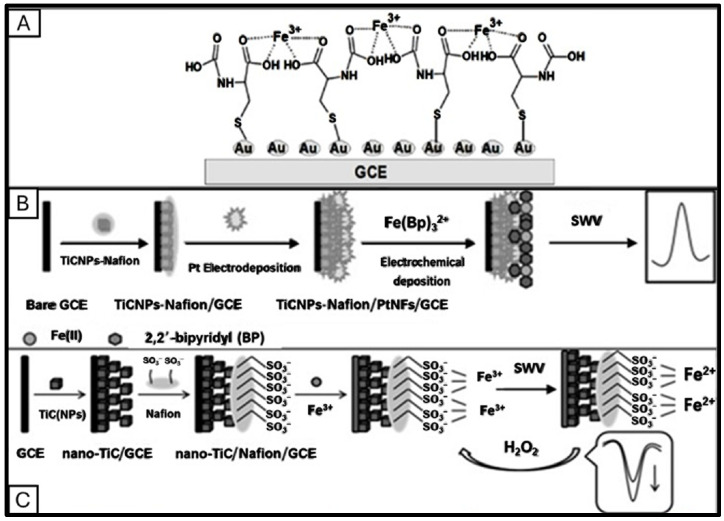
Electrode modifications with metal-based nanomaterials: (**A**) Interaction of Fe(III) with a short chain self-assembled monolayer of N-carboxyl-L-cysteine (NCLC) on a gold nanoparticles-modified glassy carbon electrode (GCE). Reproduced with permission from [60]. (**B**) Electrodeposition of platinum (Pt) nanoflowers and a complexing agent (2,2′-bipyridyl) on a GCE modified by titanium carbide nanoparticles (TiCNPs) and Nafion enhanced Fe(II) detection by SWV. Reproduced with permission from [61]. (**C**) Deposition of titanium carbide (TiC) nanoparticles and nafion on the GCE to detect Fe(III) in the presence of H_2_O_2_. Reproduced with permission from [110].

Gold-based nanomaterials are extensively applied as electrochemical sensors and biosensor modifiers. These materials are available in various forms, including gold nanoparticles (AuNPs) [60,81,111,113], nanoflowers [128,129], nanorods [130,131], and nanodendrites (AuNDs) [89,112]. AuNPs, with their two-dimensional nanostructures and high surface-to-volume ratio, serve as excellent scaffolds for fabricating electrochemical, biological, optical, and electronic sensors [132]. Due to unique quantum size effects, they offer exceptional conductivity, biocompatibility, and high electron transfer rates [91,133,134]. Figure 2A shows that a short chain self-assembled monolayer of N-carboxyl-L-cysteine (NCLC) on a glassy carbon electrode modified by electrodeposition of AuNPs achieved a remarkable detection limit of 0.03 nM for Fe(III) [60]. NCLC plays a dual role as both a stabilizing and binding agent that enhances the selectivity and sensitivity of the sensor for iron detection. Electrodeposited gold nanodendrites (AuNDs) are another prominent gold-based nanomaterial featuring highly branched, dense, long, and 3D nanostructures. These structures enlarge the electrode surface area for electroanalytical detections of iron [112]. An electrochemically deposited AuNDs on a rGO-modified GCE with a nafion coating exhibited outstanding selectivity in Fe(III) detection with less than 5% interference from competing ions [112]. Co-electrodeposition of AuNPs and rGO as a nanocomposite on a GCE showed an LOD of 3.5 nM for Fe(III) [113]. Additionally, the electrodeposition of dendritic Au nanostructures (DAuNs) on graphene oxide resulted in a lower LOD and nearly fivefold increase in the cathodic peak current for Fe(III) [89].

Furthermore, paper-based screen-printed electrodes modified with carbon black and drop-casted with nafion and AuNPs showed high conductivity and sensitivity for detecting Fe(III) in acidic solutions. This sensor achieved a detection limit of 0.9 µM. It was validated on acid-pretreated human serum samples, demonstrating a strong correlation with results from calorimetric and atomic absorption spectroscopy (AAS) methods [29]. Table 4 highlights how gold-based nanomaterials enable iron LOD in the nanomolar to micromolar range. Including catalytic reagents (e.g., H_2_O_2_) or iron-binding ligands in the samples can lower the LOD to the picomolar range. These findings underscore the versatility and effectiveness of gold-based nanomaterials in developing advanced iron electrochemical sensors.

Platinum-based nanomaterials have been integrated into alternative substrates to provide cost-effective and scalable solutions while preserving platinum’s exceptional electrochemical properties. For instance, platinum nanostructures deposited on glassy carbon electrodes reduced material usage and cost while retaining high sensitivity and electrocatalytic efficiency [61]. Silicon wafer substrates were used to fabricate platinum nanoparticles, enhancing structural stability and scalability [114,115]. Additionally, platinum nanoflowers (PtNFs) grown on titanium carbide nanoparticles (TiCNPs) were used to create a 3D architecture on a GCE, as shown in Figure 2B [61]. This nanostructure significantly enhances the anodic peak current for Fe(II) detection and achieves a low LOD of 30 pM [61]. Nanoscale titanium carbide, a cubic-phase nanoparticle, serves as a growth template, offering a high electron transfer rate and a large active surface area (Figure 2C) [110,135]. Titanium carbide (TiC) is widely used in composite materials as a reinforcing agent and is valued for its exceptional mechanical strength, unique electrical properties, and high-temperature stability [136].

Metal oxide nanomaterials, such as zinc oxide (ZnO) nanorods, have been extensively applied in developing voltammetric and potentiometric iron sensors [126]. ZnO nanorods are characterized by their direct wide bandgap (3.37 eV at 300 K) and relatively large exciton binding energy (60 meV), contributing to their exceptional electrochemical properties. Their 3D nanostructures grown on a nickel foam substrate can enhance potentiometric sensor performance [88]. It was found that their functionalization with Fe(III)-selective ionophores and other additives can improve Fe(III) adsorption, response time (10 s), and detection sensitivity with a stable Nernstian behavior and 41 mV/decade slope [88]. These innovations highlight the potential of ZnO-based sensors for selective and reliable Fe(III) detection and paving the way for further innovation in electrochemical sensing technologies.

Bismuth-based nanomaterials have become low-toxic alternatives to mercury-based sensors due to their low toxicity, electrochemical stability, and favorable performance in faradaic and non-faradaic analyses. These nanomaterials enhance Fe(III) detection by facilitating effective preconcentration through metal–ion interactions. Bismuth’s ability to form low-temperature alloys with metal ions facilitates the nucleation process during the preconcentration step, resulting in high detection sensitivity comparable to mercury-based electrodes [100]. Notably, bismuth-based electrodes are primarily insensitive to dissolved oxygen, which makes them ideal for complex sample matrixes in real-world applications [100].

Bismuth film electrodes, including tin–bismuth alloy wires, can offer a highly sensitive Fe(III) detection platform, achieving detection limits as low as 0.2 nM through the CSV method. This high sensitivity was attributed to the film’s ability to facilitate Fe(III) complex formation with a 1-(2-piridylazo)-2-naphthol ligand, enhancing signal response [50]. These sensors effectively quantified total iron in coastal rivers and seawater samples. Moreover, co-electrodeposition of bismuth with ferromagnetic nanoparticles enabled the development of magnetized carbon nanotube-based electrodes for lab-on-a-chip platforms, which achieved ultralow LODs of 0.01 nM for Fe(III) by DP-CSV at a very low peak potential (−1.25 V) [64]. However, some ambiguities remained in peak identification and characterization for Fe(II), Fe(III), iron hydroxides, and oxides, as well as in managing high holding potentials (~1.5 V) applied during analysis [64].

In another approach, bismuth nanosheets were modified with graphene oxide and deposited onto glassy carbon electrodes for Fe(III) detection at +0.6 V using DP-AdSV [59]. This method leveraged the catalytic effects of potassium bromate (KBrO_3_) and nafion as a dispersing agent for bismuth. Additionally, non-electrochemical preconcentration steps were employed to enhance Fe(III) adsorption on the electrode surface and significantly amplified current signals with LOD of 2.3 µM at 0.01–20 µM range [59]. Compared to bismuth nanomaterials, generating bismuth microrods on glassy carbon electrodes effectively addressed challenges related to bismuth hydrolysis and instability at positive potentials [137]. These microrods exhibited a superior performance than bismuth nanoparticles for voltammetric detection of iron [137]. Bismuth microrods-modified electrodes showed the merits of the metallic bismuth and bismuth oxide and directly reduced Fe(III) at approximately (+0.6 V) and achieved 6.4 nM LOD at 0.02–10 µM calibration range. These sensors were applied to distinguish various iron fractions in coastal waters, such as particulate and total dissolved iron [138].

Employing bimetallic nanoparticles in metallic-based nanomaterials’ structure is another practical approach to benefit from the properties of two metals and their synergistic effects. This improves the nanomaterial stability, sensitivity, and electrocatalytic activity compared to monometallic counterparts. Gold–bismuth nanoparticles were utilized for Fe(III) detection at micromolar levels [90], which benefited from superior catalytic and dielectric properties [139]. Bimetallic nanomaterials are often supported on host matrixes such as carbon paste, conducting polymers, or graphene oxide, which offer additional benefits like low background current and high active surface area. These composites ensure high sensitivity and stability, which makes them ideal for iron detection in complex environments [140,141]. Integrating bimetallic nanoparticles into advanced sensor platforms has further expanded the scope of iron detection technologies.

#### 4.5.3. Silica-Based Materials

Ordered mesoporous silica materials have gained significant attention in sensor development due to their high porosity and uniform pore sizes, which enhance ion transport and facilitate preconcentration of analytes at the electrode surface. These materials can be synthesized using surfactants in a sol–gel process with precursors such as tetraethyl orthosilicate (TEOS) [142]. Vertically ordered mesoporous silica films (VMSF) have gained particular attention for their exceptional properties. These films are fabricated through electrochemically assisted self-assembly (EASA) and Stöber solution growth [26,143]. VMSF are highly effective as preconcentration materials for electrochemical detection, particularly of metal ions like Cu^2+^ [144,145], Ag^+^ [146], Hg^2+^ [145], Pb^2+^ [144,147,148], and Cd^2+^ [144,147,148]. VMSF exhibits vertical channel structures with negatively charged walls, high porosity, and uniform pore size, making them ideal for designing electrochemical sensors capable of analyzing various analytes in complex samples. These include organic pollutants [149], biomolecules [150,151,152,153], drug molecules [154,155], and metal ions [26,144,145].

**Figure 3:**
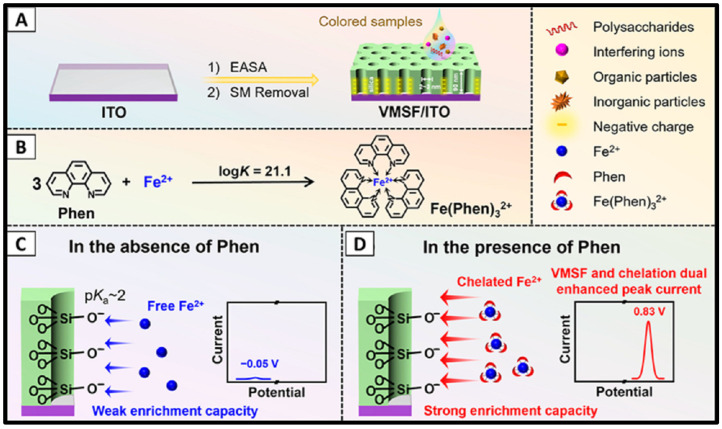
Electrode modification with silica-based nanochannels: (**A**) Modification of indium tin oxide (ITO) with vertically ordered mesoporous silica films (VMSF) using electrochemically assisted self-assembly (EASA) method, followed by removal of surfactant micelles (SM), (**B**) Chelation reaction of Fe(II) with *o*-phenanthroline (Phen), and sensing of Fe(Phen)_3_^2+^ complex, (**C**) without, and (**D**) with *o*-phenanthroline. Reproduced with permission from [26].

Recently, Huang et al. advanced this field by fabricating a vertically ordered mesoporous silica film on an indium tin oxide (ITO) substrate using the EASA method. This film was employed for the detection of Fe(II) in a complex form with *o*-phenanthroline (Fe(Phen)_3_^2+^) using DPV, as seen in Figure 3A–D [26]. The VMSF showed exceptional structural properties, including hexagonally packed nanopores with a uniform diameter of 2.6 nm, a high pore density of 7.8 × 10^12^ cm^−2^, and a porosity of 42%. These structural features remained intact before and after Fe(II) detection, ensuring high durability. Fe(Phen)_3_^2+^ was effectively preconcentrated at the electrode surface through mechanical stirring. This approach avoided the drawbacks of anodic stripping voltammetry, such as alloy formation and signal distortion caused by cathodic reduction followed by anodic stripping. This sensor exhibited a linear detection range from 1 nM to 13 μM and achieved a LOD of 0.66 nM [26]. The modified electrode was successfully applied to detect Fe(Phen)_3_^2+^ in ferrous sulfate tablets, pond and tap water, and colored samples [26]. This study highlighted the immense potential of silica-based nanomaterials, particularly VMSFs, in designing robust and sensitive electrochemical sensors for iron detection in complex matrixes.

#### 4.5.4. Conducting Polymer-Based Nanomaterials

**Figure 4:**
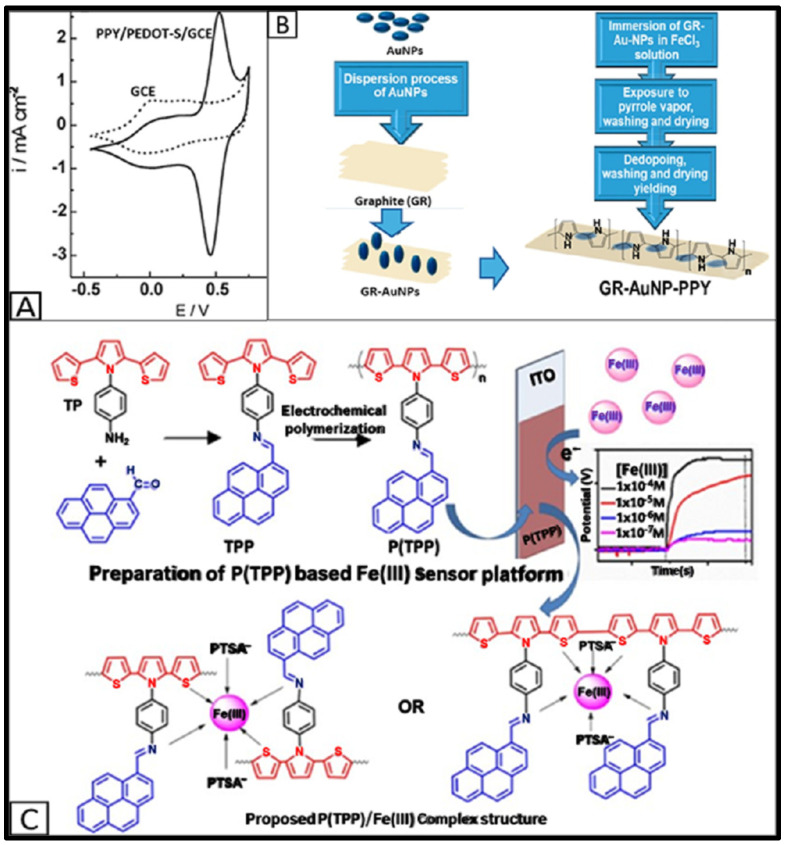
Electrode modifications with conducting polymers: (**A**) CV of Fe(III) (black line) versus background scan (dashed line) using a GCE modified with an electroconductive hydrogel composed of PPY and alkoxysulfonated PEDOT (PEDOT-s). Reproduced with permission from [31]. (**B**) Green synthesis of a hybrid nanocomposite made of AuNPs-decorated graphite and polypyrrole for ultrasensitive detection of Fe(II). Reproduced with permission from [81]. (**C**) Electropolymerisation of pyrene-substituted poly(2,5-dithienylpyrrole) P(TPP) on ITO and proposed structure of P(TPP)-Fe(III) complex in the presence of p-toluene sulfonic acid (PTSA). Reproduced with permission from [163].

Conducting polymers are excellent candidates for electrochemical sensor modifications due to their intrinsic conductivity, tuneable surface chemistry, and ability to form selective binding sites for iron ions, making them effective in complex matrixes [156,157,158,159,160]. Polymers such as polypyrrole (PPY), polyaniline (PANI), and poly(3,4-ethylenedioxythiophene) (PEDOT) are particularly promising for detecting iron ions due to their ability to form conductive networks and create unique nanostructures [9,81,161,162]. These materials offer significant advantages, including high sensitivity, chemical stability, and selective ion-binding capabilities, essential for accurate iron sensing in complex matrixes. Modifying a GCE with an electroconductive hydrogel composed of PPY and alkoxysulfonated PEDOT resulted in a well-defined cathodic peak for Fe(III) at 0.3 V vs. Ag/AgCl in 0.1 M HClO_4_. The cathodic peak current of Fe(III) was improved approximately seven times, higher than that of the unmodified electrode, with a LOD of 0.8 μM over a calibration range of 2.5–500 μM (Figure 4A) [31].

Hybrid composites made by combining conducting polymers with other nanomaterials enhance charge transport, expand the electroactive surface area, improve sensitivity, and reduce response time for iron detection. An ion-selective carbon paste sensor modified with AuNPs-decorated graphite and PPY through vapor polymerization detected Fe(II) within 3 s in a detection range of 1 µM–10 mM with an excellent Nernstian slope (Figure 4B) [81]. The unique iron-imprinting-like mechanism of the hybrid material can also improve the selectivity and sensitivity of detection. In work by Kindra et al., deferoxamine-doped PEDOT films and gold nanowires chemiresistors offered a low LOD of 300 pM for Fe(III) detection in high-ionic-strength aqueous solutions [162]. However, this type of sensor operates through changes in electrical resistance rather than conventional electrochemical signal measurements [162], which are beyond the scope of this review.

Thienyl pyrrole derivates electropolymerized on an ITO glass substrate and was used as a potentiometric sensor for Fe(III) detection. This sensor exhibited high stability, reproducibility (RSD < 5%), and a LOD of 173 nM in aqueous solutions (Figure 4C) [163]. Additionally, a novel approach using pyrrole electropolymerized on a screen-printed carbon electrode in the presence of sodium dodecyl sulfate (SDS) and iron sulfate produced an ion-selective polymer layer. This layer’s double negative charge, induced by sulfate anions, enabled the electrode to attract Fe(II) cations and facilitated potentiometric detection in the range of 1 µM–100 mM and DPV detection in the range of 0.001–10 µM with limits of detection as low as 0.87 µM and 0.58 nM, respectively [161]. The addition of SDS significantly expanded the sensor’s dynamic range by approximately 100 times compared to systems without SDS [161]. The underlying mechanism was that polyradical cations attracted sulfate anions into the polymerized film as counter ions [164]. However, the peak detected by the DPV method was very broad, potentially increasing interference from other ions in complex real-world samples. Optimizing polymer composition or integrating molecularly imprinted sites may improve selectivity and peak resolution.

Conducting polymers, alone or combined with other nanomaterials, provides robust platforms for designing advanced electrochemical sensors for iron detection. Their ability to integrate functional groups, enhance charge transfer, and form tailored nanostructures enables highly selective and sensitive detection in various samples. These characteristics make conducting polymer-based materials a basis for developing next-generation iron sensing technologies.

#### 4.5.5. Nanomaterials on Other Substrates

**Figure 5:**
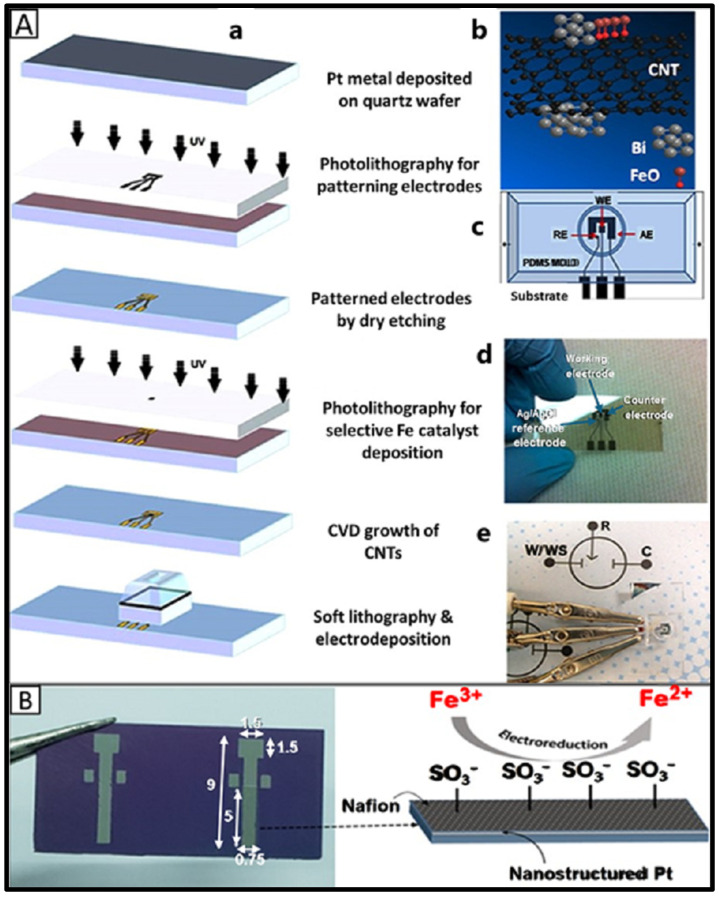
Electrode modifications on other substrates: (**A**) a–e: Fabrication process of a quartz wafer to make a lab-on-a-chip sensor by photolithography, chemical vapor deposition (CVD) of carbon nanotubes, soft lithography, and electrodeposition of bismuth (Bi) and magnetic nanoparticles (FeO). Reproduced with permission from [64]. (**B**) A silicon wafer was microfabricated with a thin film of platinum–nafion to detect Fe(III). Reproduced with permission from [115].

Integrating nanomaterials into innovative electrode substrates enhances the functionality of electrochemical sensors for iron detection. Advanced substrates, such as quartz, silicon wafers, and nickel foams, provide unique platforms for combining nanomaterials and enhancing sensing performance [64,88,114,115]. Quartz wafers, known for their chemical stability, high mechanical strength, and excellent insulating properties, are reliable substrates for nanomaterial integration and designing iron electrochemical sensors. A nanomaterial-modified quartz-based electrochemical sensor was constructed, achieving a LOD of 0.01 nM for Fe(III) and Fe(II) with a broad dynamic range (0.01 nM–10 mM), attributed to the synergistic effects of SWCNTs, bismuth and magnetic nanoparticles [64]. However, as shown in Figure 5A, the sensor’s complex fabrication process involved microfabrication, soft lithography, and electrodeposition, requiring specialized expertise and equipment. The need for cleanroom conditions and precise patterning steps increases production costs and limits scalability. Additionally, the sensor may face challenges related to long-term stability and susceptibility to interference from other metal ions, necessitating further optimization for real-world applications. Despite these limitations, the sensor’s innovative design and outstanding detection capabilities make it a promising tool for various applications.

Silicon wafers are an effective electrode substrate for fabricating platinum nanoparticles and nanograins due to their smooth surface, high conductivity, and compatibility with microfabrication techniques [114,115]. However, like quartz wafers, the fabrication process is very complex. In one approach, a platinum–nafion thin film was microfabricated on silicon wafers, followed by spin-coating a nafion layer as seen in Figure 5B. Nafion played a dual role: (1) stabilizing the electrode surface by preventing delamination of the modified layer and (2) improving iron preconcentration via its negatively charged sulfonate groups, which facilitated Fe(III) accumulation through the Donnan effect [165,166]. This sensor detected Fe(III) in water by SWV with a low LOD of 0.31 ppb (~5.6 nM) without requiring complexing agents [115]. The stable and low background current, along with the excellent reproducibility of the sensor, enabled low LOD. However, real sample validation was performed at concentrations 100 times higher than the LOD (0.31 ppb), suggesting that matrix effects or adsorption losses may influence sensor performance at ultra-low concentrations.

In another approach, a sputtered platinum thin film was fabricated using photolithography, sputtering, and lift-off methods. The sputtered Pt nanograins were uniformly dispersed in the film, and the electrode showed good stability and reproducibility [114]. With its 3D porous structure, Nickel foam provides a high surface area and excellent electrical conductivity, making it an ideal substrate for nanomaterial deposition in iron electrochemical sensing [88]. Growing seedless ZnO nanorods on a nickel foam can form highly dense, vertically aligned single crystals, functionalized with a selective Fe(III) ionophore and additional membrane components. The developed ion-selective electrode enabled a low LOD (1 µM) in a wide detection range (0.005–100 mM) within less than 10 s [88].

### 4.6. Potentiometric Ion-Selective Electrodes

Potentiometric sensors possess several advantageous characteristics that make them suitable for on-site or real-time monitoring. These sensors are relatively easy to manufacture in various sizes, including micro-sized formats, and are simple to operate for extended periods with minimal intervals between measurements [167]. Potentiometric sensors consist of an ion-selective electrode (ISE) and a reference electrode. ISEs are designed as indicator electrodes for detecting specific ions [168,169]. ISEs are characterized by a relatively wide linear operational range, which enables Nernstian response to the primary ion activity in the solution. Unfortunately, without specific modifications to the sensor design or the measurement method, ISEs do not achieve sufficiently low detection limits to effectively detect trace levels of iron below 0.1 µM concentrations. ISEs often require sample pre-treatment steps, such as acidification and selective ion removal, to enhance performance. The response of ISEs to the activity of metal ions depends on the detection of ionized species, which necessitates careful pH adjustment to ensure accurate measurements [169]. ISEs have not yet been widely adopted for routine metal ion monitoring due to practical challenges, including the need for frequent calibration, limited resolution for detecting divalent or multivalent ions, constrained detection ranges, slow response times, limited capability for multi-ion sensing, low operational stability, high membrane resistance, and interference from other ions [170,171]. To address these issues, several strategies were explored, such as modifying electrode designs, pre-treating electrodes, and employing controlled measurement methods. These approaches aim to achieve higher sensitivity and lower detection limits for iron detection across different types of ISEs, including solid-state [88,172], conventional, and solid-contact configurations, as reviewed in Table 5. Conventional ISEs, such as KCl, employ internal solutions to facilitate ion transport. However, challenges such as calibration needs, internal solution refilling, and maintenance restrict their applications to controlled laboratory settings. This limits their use in portable or point-of-care (POC) systems [73,79,173]. Efforts to overcome these limitations include replacing internal solutions with solid-state materials capable of ion-to-electron signal transduction to make calibration-free and miniaturized devices [174,175].

Solid-state ISEs utilize solid ion-selective membranes by often incorporating materials like metal oxides to improve sensitivity and stability. Metal oxides, comprising positive metal cations and negative oxygen ions, exhibit distinctive optical and electrical properties. Hematite (α-Fe_2_O_3_) nanoparticles, in particular, stand out for their affordability, eco-friendliness, high stability, semiconducting properties, and magnetic behavior [176,177,178,179]. Paut et al. developed a solid-state ISE by synthesizing hematite nanoparticles together with polycrystalline silver sulfide, which showed superior potentiometric responses compared to other nanoparticles, such as magnetite (Fe_3_O_4_), boehmite (γ-AlO(OH)), and alumina (Al_2_O_3_) [172]. The hematite-modified electrodes demonstrated improved Nernstian slopes for Fe(III) detection without using ionophores through enhancing ion exchange properties with 30 s response times and one-month operational stability [172].

Solid-contact ISEs eliminate the need for internal solutions by incorporating a solid transducer layer beneath the polymeric ion-selective membrane. This design enhances the selectivity, stability, and reliability of potential responses while reducing ion flux from membranes [72,171,172,180]. Strategies to improve solid-contact ISE performance include reducing membrane resistance through increased surface area or using thin-layer ion-selective membranes [170]. Advanced materials such as carbon materials, conducting polymers (e.g., polypyrrole, polyaniline, poly(3-octylthiophene)), and hydrogels are widely employed in sensor technology for their unique chemical, optical, and electrical properties [181,182]. Kumar et al. enhanced electrode conductivity and surface roughness by incorporating 1% MWCNTs into the electrode membrane [78].

**Figure 6:**
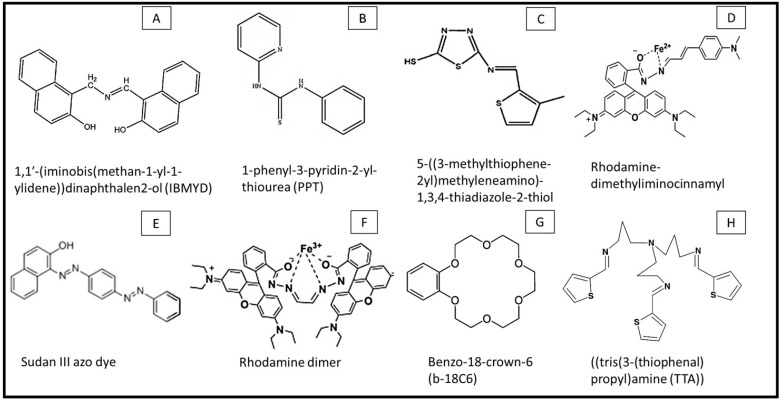
Structure of ligands and ionophores used in ion-selective electrodes. (**A**) Adopted from [73], (**B**) [173], (**C**) [191], (**D**) [77], (**E**) [25], (**F**) [76], (**G**) [74], (**H**) [79].

Ion-selective membranes in ISEs comprise an ion carrier, ion exchanger, plasticizer, and polymer matrix. Plasticizers play a crucial role in enhancing the physical properties of the membrane by improving its plasticity and fluidity. Their polarity affects the dielectric constants and lipophilicity of the membrane components, which is crucial for ensuring compatibility with the ion carrier and achieving optimal selectivity [182]. Common plasticizers, such as tricresylphosphate (TCP), bis(2-ethylhexyl) sebacate (DOS), dibutyl phthalate (DBP), bis(2-ethylhexyl) phthalate (DOP), sodium tetraphenylborate (NaTPB) and 2-nitrophenyl octyl ether (NOPE), etc., are incorporated in PVC membranes of iron potentiometric sensors to improve membrane workability, operational concentration range, shelf life, and sensor stability [183]. Plasticizers should possess specific properties, such as high molecular weight, low exudation tendency, high lipophilicity, adequate dielectric constant and viscosity, low vapor pressure, and high capacity to dissolve additives to ensure their effectiveness in membrane applications [183]. Incorporating ionophores into ISE membranes is another approach to enhance iron selectively [12]. Ionophores act as carriers facilitating ion exchange and efficient signal transduction from an aqueous sample phase into the polymeric membrane phase by forming complexes with target ions [184]. Table 5 and Figure 6 shows a range of ionophores applied in potentiometric sensors to detect iron ions, such as benzo-18-crown-6 (B-18C6), bis-bidentate Schiff (BBS), µ-bis(tridentate), 2-[(2-hydroxy-1-propenyl-buta-1,3-dienylimino)methyl]-4-p-tolylazo-phenol (HPDTP), 4-amino-6-methyl-3-methylmercapto-1,2,4-triazin-5-one (AMMTO), N-(2hydroxyethyl)ethylenediamine-N, N′, N″-triacetic acid (NTA) [74], (methyl 6-(hydroxymethyl) picolinate) [185], 2,6-bis-(carboxamide methyl ester)pyridine derivative [186], and (*E*)-3-((2-aminoethylimino)methyl)-4*H*-chromen-4-one (IFE) [78]. Schiff bases are among the essential ligands due to their rapid exchange kinetics and well-known coordination capabilities [12], which act as efficient donor–bridge–acceptor systems [187,188]. Ionophore-based ISEs provide a different selectivity pattern toward ion detection than ion-exchange-based ISEs. Their selectivity is based on the free energy of ion transfer from the aqueous solution to the membrane phase, the complex formation constant between the extracted ions and the ionophore, and concentrations of the active membrane components [189].

PVC membranes can significantly affect the selectivity and sensitivity of ISEs. While they are commonly used for ISEs due to their flexibility and compatibility, they face challenges like short shelf life, difficulty in miniaturization, being time-consuming, and inconsistent manual fabrication methods [190]. Hence, future research should focus on developing alternative membrane materials, improving miniaturization techniques, improving sensor stabilities, and extending detection capabilities.

**Table 5:** Potentiometric ion-selective electrodes used for the detection of iron.

Electrode	Iron Species	Ligand/Ionophore	Method	Calibration Range	LOD	Reference
Phosphotungstate/TCP/PVC/SPCE	Fe(III)	Phosphotungstate	Potentiometry	0.1 μM–25 mM	0.16 μM	[190]
PVC/*o*-NPOE/NaTPB/graphite electrode	Fe(III)	L_2_	Potentiometry	0.67 μM–100 mM (polymeric membrane electrodes (PMEs) with L2)	0.14 μM (PME with L2)	[191]
				83 nM–100 mM (graphite electrode)	23 nM (graphite electrode)	
PVC/*o*-NPOE/graphite electrode	Fe(II)	RC	Potentiometry	0.1 μM–100 mM	74 nM	[77]
PVC/graphite electrode (PVC/DOS/NaTPB/RD)	Fe(III)	RD	Potentiometry	0.1 μM–100 mM	47 nM	[76]
PVC/CFMEPI/KTCIPB/ioctyl sebacate/copper wire	Fe(III)	CFMEPI	Potentiometry	1 μM–10 mM	0.6 μM	[75]
PVC/*o*-NPOE/IFE/NaTPB/MWCNTs/GCE	Fe(II)	IFE	Potentiometry DPV	0.1 μM–100 mM	25 nM	[78]
				0.99–29 μM	61.3 nM	
Ionophore/carbon/TCP	Fe(III)	Sud III azo dye	Potentiometry	0.01 μM–10 mM	0.01 μM	[25]
SPCE (ionophore/plasticizer/PVC/carbon)				0.0025 μM–10 mM	0.0025 μM	
PVC/DOS/Fe(II) phthalocyanine/KTpCIPB	Fe(III)	Fe(II) phthalocyanine	Potentiometry	1 μM–0.1 M	0.2 μM	[192]
B-18C6/PVC/*o*-NPOE/KTpClPB	Fe(III)	B-18C6	Potentiometry	1 μM–100 mM	0.8 μM	[74]
Graphite powder/ionophore/paraffin oil/carbon paste electrode	Fe(III)	Schiff-based ionophore	Potentiometry	0.1 μM–10 mM	0.05 μM	[80]
α-Fe_2_O_3_ NPs/Ferric phosphate/Ag_2_S/PTFE/epoxy plate electrode body	Fe(III)	None	Potentiometry	1.2 μM–10 mM	1 μM	[172]

## 5. Pretreatment of Samples

The complex matrixes of real samples often necessitate pretreatment methods, such as wet digestion, precipitation, acidification, and dilution, to minimize interference and ensure accurate iron detection. Appropriate sample collection and storage are also crucial for maintaining integrity. Table 6 summarizes the pretreatment strategies used for iron electrochemical sensors across various sample types. Common pretreatment approaches involve using specific chemicals to extract iron and minimize interference from other substances in complex sample matrixes. Acid digestion facilitates iron release, where acids like nitric and hydrochloric acid break down biological and food samples. Chelating agents such as DTPA prevent oxidation, reduce agents (e.g., ferrocyanide and hydroxylamine), and convert iron to its more detectable Fe(II) form, improving sensitivity. Conversely, oxidizing agents such as hydrogen peroxide and potassium bromate help to break down organic complexes, release bound iron, and remove interfering substances [49,51]. Sample dilution with buffers or acid solutions mitigates matrix effects. It improves signal clarity by providing a suitable supporting electrolyte, while pH adjustments stabilize iron ions by preventing hydrolysis, which can otherwise affect the accuracy of measurements.

For biological fluids (blood, plasma, serum, urine, saliva), acid digestion followed by filtration is commonly used to remove proteins, lipids, and cells that can interfere with detection. High-temperature acid digestion is effective in denaturing iron-binding proteins and was applied across various sample types, including biological fluids, non-biological, and food samples [12,30,73,193]. The most frequently used acids are concentrated nitric acid, hydrochloric acid, trichloroacetic acid, and diethylenetriaminepentaacetic acid (DTPA), as detailed in Table 6. Alternative methods, such as UV lamps and microwave-assisted digestions, help extract iron from organic iron–ligand complexes in water and environmental samples, which enable the analysis of total dissolved iron [50,138]. The choice of pretreatment depends on the target iron species and the need to enhance sensitivity while minimizing interference. While reducing and oxidizing agents improve detection, some studies have explored direct detection methods without pretreatment to align with the development of rapid diagnostic tools with point-of-care (POC) applications. Simplifying pretreatment enhances the practicality of electrochemical sensors for real-world use, but it poses challenges in addressing matrix interferences. While some studies demonstrated the direct detection of iron ions without pretreatment, these methods typically targeted higher analyte concentrations, where interference is less pronounced. Ghoneim (2010) and Merli et al. (2014) detected iron at nanomolar concentrations using cathodic stripping voltammetry (CSV) without requiring pretreatment [58,63]. Similarly, Liu and Wang (2014) quantified Fe(III) in seawater samples by adsorptive stripping voltammetry without pretreatment [60], and Ali et al. (2019) reported the detection of spiked samples in the tens of nanomolar range without additional processing [25]. In other cases, potentiometric methods allowed direct measurement of iron in water and pharmaceutical samples without sample pretreatments [25,75,81,163,190]. However, direct detection methods without pretreatment may not be universally applicable, especially at lower analyte concentrations where interference becomes a significant concern. In the case of biological samples, further interference comes from macromolecules such as proteins, lipids, and organic compounds that naturally bind metal ions and reduce the ions’ availability for electrochemical detection. Interfering small molecules such as low-molecular-weight antioxidants, vitamins, salts, coexisting ions, and metabolites can also compete with iron ions and cause nonspecific interactions. These necessitate efficient sample pretreatments to minimize competing signals, prevent electrode fouling, enable trace detection, and stabilize the target metal ion species. Thus, future advancements should balance sensitivity and simplicity to allow robust iron detection in complex matrixes with minimal sample preparation.

**Table 6:** Pretreatments applied to prepare actual samples before iron analysis.

Iron Species	Samples Tested	Pretreatment Strategies	Reference
Fe(III)	Certified reference riverwater	UV digestion (2 h, 150 W)	[194]
Fe(III)	Seawater, Synthetic seawater Certified reference material (CRM) samples (i.e., CRM-mixed food diet, CRM-seawater, quality control standards)	Filtration, acid digestion, heating, dilution, pH-adjusted to 4	[57]
Fe(III)	Ground, tap, and bottled natural water samples	None Dilution by acetate buffer (pH 5) and the addition of 5-Br-PADAP ligand solution	[58]
Fe(III)	Biological standard reference materials (pepperbush, human hair, mussels, and pond sediment). Non-biological samples (tap water, mineral water, and wastewater)	Acid digestion by nitric acid (1 g:5 mL), dilution, filtration, and dilution again.	[73]
Fe(III)	Tap water, river water, wastewater, iron tablet	None	[173]
Fe(II)	Lentil, wheat seed, and barely seed	Acid digestion by nitric acid (2M), sonication (60° C, 15 min), NaOH (0.1 M) addition, hydrazine solution (0.01 M) addition, filtration, and dilution.	[49]
Fe(III)	River water, wastewater	Acid digestion by H_2_O_2_ (1N) and HNO_3_ (1N), dilution, and pH-adjusted to 3.	[79]
Fe(III)	Lixiviated aqueous solution of polluted soil	None	[9]
Total iron	Water samples (tap water, well water, river water, stratal water, petroleum well water, pore water, wastewater, swampy water)	Evaporation by nitric acid (110–120 °C), heating the residue (450 °C, 20–30 min), dissolving in HCl (1:1), evaporation at 100–120 °C, and dissolving the residue in HCl and water. An aliquot of the solution was finally used with HCl as a supporting electrolyte.	[193]
Fe(III)	Drinking water (commercially bottled natural mineral water)	Water samples were acidified with HNO_3_ and KNO_3_.	[94]
Fe(III)	(1) Ironorm capsule (2) Venofers ampoule (3) V.M. protein powder (4) Corn leaves (5) Ferrotitanium alloy	(1) Capsules: powdering and dissolving in HNO_3_, adjusted pH to 2, dilution. (2) Ampoule: Dilution of contents (3) Protein powder: Acid digestion by trichloroacetic acid (TCA) and dilution. (4) Corn leaves: Heating and making ash in a furnace, acid digestion by HCl, heating, filtration, and dilution. (5) Ferrotitanium alloy sample: Acid digestion by HCl, heating, filtration, adding NaF to mask Al(III) by F^−^, and dilution.	[30]
Fe(III)	(1) Water samples (2) Soil samples (3) Fish tissue samples	(1) Water samples: adjusted pH to 2.5. (2) Soil samples: drying, mixing with diethylenetriaminepentaacetic acid, filtration, and adjusting pH to 2.5. (3) Fish tissue samples: acid digestion and adjusted pH to 2.5.	[183]
Fe(III)	Polluted water samples (formation, tab/sea/river waters)	Adjusted pH to 3.	[190]
Fe(III)	River and wastewater, soil, apples, vegetables (potato, brinjal, spinach), and medicinal plants (e.g., *Adhatoda vasica* (Arusa), *Ocimum sanctum* (Tulsi), *Withania somnifera* (Ashwagandha) and *Cassia fistula* (Amaltas))	(1) Soil samples: acid-digestion, heating, filtration, and dilution. (2) Water samples: acid digestion, pH-adjusted to 5. (3) Apple and vegetable samples: washing, cutting, making ash (200–500 °C, 5 h), washing, heating (10 min), filtration, and dilution. (4) Medicinal plants: drying, powdering, acid-digestion, heating, HCl addition, heating, filtration, neutralizing the filtrate by NH_3_, and dilution.	[191]
Fe(III)	Red wine	Dilution by KCl (0.5 M) and HCl (0.05 M)	[71]
Total iron	Snow, tap water, synthetic seawater, natural seawater	None	[63]
Fe(III)	Seawater	None	[60]
Fe(II)	Synthetic water samples Ferrous sulfate tablets Iron syrup	Filtration, adjusted pH (1.5–6.5) by nitric acid or hexamine. Tablets: powdered, dissolved in nitric acid and HCl (1:3), shaken, heated, diluted, and filtered.	[77]
Fe(III)	Synthetic water samples Venofer iron injection Iron dextran injection	Filtration, pH adjustment (1.5–6.5) by nitric acid or hexamine.	[76]
Fe(III)	Coastal seawater Coastal river water	Filtration and storage, UV digestion (pH < 2, 30 min), then dilution	[110]
Total dissolved iron	Coastal water River water	Filtration and storage, then, adjusted pH (pH < 2) by HCl (30%) and H_2_O_2_ (30%), followed by UV digestion by 500 W UV lamp (30 min)	[111]
Total dissolved iron	Coastal river water and seawater	Filtration and storage, then UV-digestion (500 W, 30 min, at pH < 2)	[50]
Total dissolved iron	(1) Sea sediment pore waters (2) Coastal river water and Coastal seawater	(1) Pore water samples: centrifuging (3000 rpm, 15 min), acidifying to pH < 2, storing at 4 °C, then dilution by HCl (10×) (2) Coastal water samples: filtration, storing at 4 °C, and dilution by HCl (10×)	[112]
Fe(III)	(1) Tap and river waters (2) Ferimax syrup (Fe(III) hydroxide complex polymaltose)	(1) Water samples: none (2) Syrup samples: dilution (4000-fold)	[75]
Fe(II)	Coastal seawater	Nitrogen purging, filtration, dilution, and then adding to acetate buffer (pH 4.5)	[61]
Fe(III)	Coastal river waters	Filtered and stored at 4 °C, pH was adjusted by HNO_3_ and H_2_O_2_ to less than 2.0, digestion by a 500 W UV lamp, and dilution by HCl as a supporting electrolyte.	[59]
Fe(II) Fe(III)	(1) Iron supplement tablets (2) Red wine	(1) Tablets: dissolving in water by sonication and centrifuging (4000 rpm, 20 min). (2) Red wine: dilution by HCl and KCl	[195]
Fe(III)	Coastal waters	Filtration, storing at 4 °C, then UV digestion by UV lamp (500 W), and dilution by acetate buffer (pH 6)	[113]
Fe(III)	Coastal river waters	Filtration, acidification, storing at 4 °C, then adding H_2_O_2_, UV digestion by 500 W UV lamp, and dilution by HCl.	[89]
Total dissolved iron	Coastal waters	Filtration, stored at 4 °C, then adjusted pH by HNO_3_ and H_2_O_2_ to less than 2.0, UV digestion by a 500 W UV lamp, and dilution (10×) with HCl.	[137]
Total iron	(1) Tap and river waters (2) Certified reference surface water	(1) Filtration, UV digestion (2 h, 400 W Hg lamp), addition of H_2_O_2_ (30%, 0.01 mL) and HCl (36%, 0.01 mL) (2) Certified reference surface water did not need pretreatment and mineralization by UV lamp.	[106]
Fe(III)	Tap water	Mixing with HCl	[54]
Total iron and acidified dissolved iron	Local coastal river water	(1) For total iron: acidification, filtration, UV digestion, and dilution by acetate buffer (pH 6). (2) For dissolved iron: acidification, filtration, and dilution by acetate buffer (pH 6).	[62]
Fe(III)	Blood serum	Mixing with trichloroacetic acid (20%), heating, sonicating, centrifuging (10 min, 10,000 rpm), and adjusting pH to 7.0.	[12]
Fe(III)	Tap water	None	[163]
Fe(III)	Formation water, tap water, river water, cooling tower water, and wastewater	None	[25]
Fe(III)	Local well Local tap water	Filtration, UV digestion, and pH adjustment to 1 by adding HCl.	[114]
Fe(III)	Drinking tap water and hospital wastewater	Treating by electrocoagulation process and adding a mixture of HNO_3_ and H_2_O_2_.	[74]
Fe(II)	Spinach samples	Cleaning, washing, cutting leafy parts, storing, then drying (60 °C, for 48 h), acid digestion, and adding a reducing reagent (hydroxylamine).	[105]
Fe(III)	(1) Local tap water (2) Local well	(1) Tap water: digestion by H_2_O_2_ and UV lamp digester (20 min) and adding 0.1 M HCl. (2) Well water: dilution (10×) before adding to HCl.	[115]
Total iron, total dissolved iron, and particulate iron	Coastal river waters	(1) For total iron: acidification by HCl (1.8 pH, 24 h) to release organic matter complexed iron and particulate iron, filtration, and storing at 4 °C, then dilution by HCl buffer with an addition of potassium bromate (oxidizing agent). (2) For total dissolved iron: Filtration, acidification by HCl, storing at 4 °C, then dilution by HCl buffer with an addition of potassium bromate (oxidizing agent). To detect particulate iron: No pretreatment (total dissolved iron value was subtracted from total iron value)	[138]
Fe(III)	Lake water Seawater	Filtration, digestion by microwave, dilution (10×), and adding to 0.1 M HCl.	[90]
Fe(II)	Tap water (pure and spiked form)	None	[81]

## 6. Conclusions

Monitoring iron levels in environmental, biological, food, and drink samples ensures quality, safety, and health. Electrochemical sensors offer a promising alternative to traditional techniques such as ICP-MS and ICP-OES by providing a portable, cost-effective, and rapid solution. Over the past decade, significant progress has been made in developing mercury-free electrochemical sensors for metal ion detection [87,196,197,198], driven by advancements in electrode modifications using nanomaterials, selective ligands/ionophores, conducting polymers, biopolymers (polydopamine), ion-exchange membranes, and hybrid composites [25,30,64,81,90,109,115,116]. These innovations have expanded sensors’ sensitivity and selectivity, enabling iron detection at trace levels in complex matrixes. Additionally, advancements in sample pretreatment methods have helped mitigate interferences from real-world sample matrixes, although some sensors have demonstrated direct detection capabilities without pretreatment.

Despite these advancements, several challenges remain. Modified electrodes often suffer from issues related to long-term stability, reproducibility, and performance under real-world conditions. Many studies report low detection limits in standard solutions; however, these values do not always translate effectively to complex sample matrixes where interference from other ions is prevalent. This highlights the need for comprehensive validation of sensors in real samples and the development of more robust materials and configurations. Also, the interaction mechanism of iron species and modified electrode materials at the electrode/electrolyte interface has not been well studied. The limit of detection reported in most reviewed studies did not show a correct assessment of the sensor detection capabilities. LODs are invariantly obtained in iron standard solutions without considering any interference from the sample matrix. Most studies used the formula (LOD = 3 × tandard deviation of blank/sensitivity) to calculate LOD, where evaluating the standard deviation of blank is difficult. Such a LOD value does not even warrant that the analysis is feasible in, e.g., natural waters, as several interfering ions may exceed the concentrations tolerable by the method. In some studies, validation of iron sensors was achieved at concentrations 100–1000 times higher than the LOD [63,115,191]. Moreover, potential limitations may arise, primarily associated with sensitivity, electrode preconditioning, and re-calibration steps, mainly when using potentiometric sensors. Although potentiometric sensors can be utilized for long-term in situ (in-sample) monitoring of iron, they scarcely detect and quantify multiple ions simultaneously, in contrast to voltammetric methods. Furthermore, the commercialization and mass production of modified electrodes remain underexplored, which limits their broader application and accessibility.

We expect that the limitations described above will be addressed by designing novel iron-selective ligands (e.g., peptide-based ligands, macrocyclic compounds, hydroxamic acid-based ligands), employing innovative electrode materials such as hierarchical nanostructures [199], MXenes materials [200], molecularly imprinted polymers (MIPs) [201], and metal–organic frameworks (MOFs) [202], and integrating hybrid nanomaterials to enhance sensitivity and selectivity. Emerging surface modification techniques, including atomic layer deposition (ALD) and laser-induced graphene (LIG), may improve electrode performance and durability. Additionally, minimizing sample pretreatment through microfluidic integration and employing computational approaches, such as machine learning-driven sensor optimization and in situ electrochemical modelling, could enhance detection efficiency. Cross-disciplinary collaboration will be critical in overcoming these challenges and enabling the development of cost-effective, highly sensitive, reliable, and portable sensors for diverse applications. Future advancements may lead to the integration of these sensors into point-of-care diagnostics, wearable devices, and automated monitoring systems for real-time iron detection in clinical, environmental, and industrial settings. The continued evolution of mercury-free electrochemical sensors, combined with novel materials and intelligent sensing strategies, positions them as key tools for addressing global iron-related health and environmental challenges. Research and innovation will be essential in unlocking their full potential and expanding their applications.

## Figures and Tables

**Table 4:** Electrochemical sensors modified with nanomaterials and composites for iron detection.

Electrode	Iron Species	Reagent	Method	Calibration Range	LOD	Reference
NCLC/AuNPs/GCE	Fe(III)	None	DP-AdSV	0.1–1.8 nM	0.03 nM	[60]
Nafion-CNT/GCE	Fe(III)	HNO_3_, KNO_3_	LSSV	1–50 μM	0.71 μM	[56]
Nafion/TiCNPs/GCE	Fe(III)	H_2_O_2_	SWV	0.07–70 μM	7.2 nM	[110]
AuNPs/Methylene blue/rGO/GCE	Fe(III)	None	DPV	0.3–100 μM	15 nM	[111]
Nafion/AuNDs/IL-rGO/GCE	Fe(III)	None	SWV	0.3–100 μM	35 nM	[112]
PtNFs/TiCNPs-Nafion/GCE	Fe(II)	2,2-bipyridyl	SW-ASV	1 nM–6 μM	0.03 nM	[61]
AuNPs/rGO/GCE	Fe(III)	5-Br-PADAP	DPV	30 nM–3 μM	3.5 nM	[113]
DAuN/GO/GCE	Fe(III)	H_2_O_2_	DPV	7 nM–1 μM	1.5 nM	[89]
Sputtered PtNPs/silicon wafer	Fe(III)	None	SWV	0.3–5 ppm	90 ppb (~1.6 μM)	[114]
Nafion/nano-grain Pt/silicon wafer	Fe(III)	None	SWV	1–250 ppb	0.31 ppb (~5.6 nM)	[115]
Thermally reduced graphene-nafion/PtDE	Fe(III)	None	SWV	1–200 ppb	0.08 ppb (~1.4 nM)	[116]
Piroxicam/Graphene/SPCE	Fe(III)	KBrO_3_	DPV-AdSV	1–3500 ng/mL	0.3 ng/mL (~5.4 nM)	[117]
Au-BiNPs/L-cysteine-GO/GCE	Fe(III)	None	SWV	0.2–50 μM	0.07 μM	[90]
GR-AuNPs-PPY/carbon paste	Fe(II)	None	Potentiometry	1–10 mM	0.79 μM	[81]
Alanine-polydopamine-rGO/GCE	Fe(II)	None	DP-ASV	40–120 ppb	50 ppb (~0.9 μM)	[55]
N-CQD/AgNPs/β-cyclodextrin/GCE	Fe(II) Fe(III)	None	DPV	Fe(II): 0.6–10 mM Fe(III): 0.2–10 mM	Fe(II): 0.2 mM Fe(III): 0.033 mM	[28]
Nafion/AuNPs/carbon black/paper SPE	Fe(III)	None	SWV	Up to 10 mg/L	0.035 mg/L (0.6 μM) 0.05 mg/L for serum (~0.9 μM)	[29]
N, S-GQD/GCE	Fe(III) Total Fe	KNO_3_	Amperometry SWV	1–100 nM 1–120 nM	0.23 nM 1 nM	[27]

## Data Availability

No new data were created or analyzed in this study. Data sharing is not applicable to this article.

## Abbreviations

AAS	Atomic absorption spectroscopy
AdSV	Adsorptive stripping voltammetry
AMMTO	4-Amino-6-methyl-3-methylmercapto-1,2,4-triazin-5-one
ANTNA	ω-Thio nitrilotriacetic acid derivative (nitrilotriaceticacidderivative(N-[5-[[[[20-(acetylthio)-3,6,9-trioxaeicos-1-yl]oxo]carbonyl]amino]-1carboxypentyl] iminodiacetic acid)
ASV	Anodic stripping voltammetry
B-18C6	Benzo-18-crown-6
BBS	Bis-bidentate Schiff
5-Br-PADAP	2-(5-Bromo-2-pyridylazo)-5-diethylaminophenol
CFMEPI	5-Chloro-3-[4-(trifluoromethoxy)phenylimino]indolin-2-one
CSV	Cathodic stripping voltammetry
CV	Cyclic voltammetry
DBP	Dibutyl phthalate
DAuNs	Dendritic Au nanostructures
DFO	Deferrioxamine
DPV	Differential pulse voltammetry
EASA	Electrochemically assisted self-assembly
EIS	Electrochemical impedance spectroscopy
FcMeOH	Ferrocenemethanol
HPDTP	2-[(2-Hydroxy-1-propenyl-buta-1,3-dienylimino)methyl]-4-p-tolylazo-phenol
IFE	(*E*)-3-((2-aminoethylimino)methyl)-4*H*-chromen-4-one
IL-rGO	Ionic liquid-reduced graphene oxide
ISEs	Ion-selective electrodes
ITO	Indium tin oxide
KTCIPB	Potassium tetrakis(p-choro) fenylborate
KTpClPB	Potassium tetrakis(4-chlorophenyl)borate
L_2_	5-((3-methylthiophene-2yl) methyleneamino)-1,3,4-thiadiazole-2-thiol
LSSV	Linear sweep stripping voltammetry
LSV	Linear sweep voltammetry
MPA	3-Mercaptopropionic acid
C6	6-Mercaptohexanoic acid
NaTPB	Sodium tetraphenylborate
NCLC	N-carboxyl- L-cysteine
nM	Nanomolar
*o*-NPOE	*o*-Nitrophenyloctylether
NPs	Nanoparticles
N-CQD	Nitrogen-carbon quantum dot
N, S-GQD/GCE	Nitrogen and sulfur co-doped graphene quantum dot
NTA	N-(2hydroxyethyl)ethylenediamine-N, N′, N″-triacetic acid
PEDOT-s	Poly(4-(2,3-dihydrothieno[3,4-*b*][1,4]dioxin-2-yl-methoxy)-1-butanesulfonic acid
PtDE	Platinum disk electrode
PtE	Platinum electrode
pM	Picomolar
PTFE	Polytetrafluoroethylene
PVC	Polyvinyl chloride
PAN	1-(2-pyridylazo)-2-naphthol
RC	Rhodamine-dimethyliminocinnamyl
RD	Rhodamine dimer
rGO	Reduced graphene oxide
RSD	Relative standard deviation
SAM	Self-assembled monolayer
SDS	Sodium dodecyl sulfate
SPCE	Screen-printed carbon electrode
SPE	Screen-printed electrode
SWCNT	Single-walled carbon nanotubes
SWV	Square wave voltammetry
TAC	Trichloroacetic acid
TCP	Tricresylphosphate
TEA	Triethanolamine
